# Comparing the effects of aquatic-based exercise and land-based exercise on balance in older adults: a systematic review and meta-analysis

**DOI:** 10.1186/s11556-024-00349-4

**Published:** 2024-05-19

**Authors:** Ying Deng, Zheng Tang, Zhengting Yang, Qi Chai, Wenting Lu, Yunshi Cai, Yiting Luo, Yongzhao Zhou

**Affiliations:** Sichuan University, West China Hospital, Chengdu, 610041 Sichuan China

**Keywords:** Aquatic exercise, Balance, Meta-analysis, Older adults

## Abstract

**Background:**

Balance plays a crucial role in the daily activities of older adults. Aquatic-based exercises (AE) are widely conducted as an alternative to land-based exercises (LE). Previous studies have compared AE and LE as effective ways to improve balance and have yielded inconsistent results. Therefore, this review aimed to compare the effects of AE and LE on balance function in older adults.

**Methods:**

Electronic databases, including PubMed, Web of Science, Scopus, and Embase, were searched. Randomized controlled trials published from January 2003 to June 2023 were included following predetermined criteria. Data extraction was carried out by two independent reviewers. Data synthesis was conducted using RevMan 5.3 software. The fixed-effect model or random-effect model was chosen based on the results of the heterogeneity test. Meta-analysis for the effect sizes of balance outcomes was calculated as standardized mean difference (SMD) with 95% confidence intervals (CI). The quality of the included studies was evaluated using the Physiotherapy Evidence Database (PEDro) scale. This review was registered at PROSPERO CRD42023429557.

**Results:**

A total of 29 studies involving 1486 older adults (with an average age of 66.2 years) were included. Meta-analysis results indicated that AE could improve balance ability based on two tests: the Berg balance scale (BBS: SMD = 1.13, 95% CI 0.25 to 2.00, *p* = 0.01, I^2^ = 94%) and the 30-s chair stand test (30 CST: SMD = 2.02, 95% CI 0.50 to 3.54, *p* = 0.009, I^2^ = 96%). However, there were no significant differences between the AE group and the LE group in terms of the 6-min walking test (6 MWT: SMD = 0.13, 95% CI -0.16 to 0.43, *p* = 0.38, I^2^ = 62%) and time up to go test (TUGT: SMD = 0.44, 95% CI -0.44 to 0.91, *p* = 0.07, I^2^ = 85%). Older adults with different health conditions have different gains in different balance measurements after AE intervention and LE intervention.

**Conclusions:**

Although this was influenced by participant health status, transfer effects, sample size, and other factors, AE offers better benefits than LE for improving balance function in older adults.

**Supplementary Information:**

The online version contains supplementary material available at 10.1186/s11556-024-00349-4.

## Introduction

A report from the World Population Prospects 2019 predicts that by 2050, one in six of the population in the world aged 65 and above, accounting for 16% [1], the global population aged 65 years and over is growing faster than younger groups. With the rapid increase in older adults worldwide, a number of older adults with balance dysfunction caused by aging and diseases is also rapidly growing [2, 3]. Balance impairments are a major contributing factor to falls in older adults, which in turn lead to increased mortality and disability rates [4]. According to the World Health Organization (WHO), approximately 28–35% of individuals over the age of 65 and 32–42% of those over the age of 70 experience a fall each year [5].

Balance is the ability to stay upright or stay in control of body movements, which requires not only good coordination but also good levels of other fitness components such as agility [6, 7]. Static balance is the ability to maintain postural stability and orientation with the center of mass over the base of support and the body at rest [8]. Dynamic balance is the ability to transfer the vertical projection of the center of gravity around the supporting base of support. [9]. It has become routine to use semiqualitative functional assessments such as the Berg Balance Scale (BBS) or the Timed Up and Go Test (TUGT) as a way to estimate balance function [10]. For static balance assessment, the common way is a single-limb stance with eyes closed or open [11]. Physical exercises are effective measures to improve balance and reduce the risk of falls in older adults [12, 13]. Howe et al. indicated that land-based exercises (LE) are the most common form of intervention for improving balance and reducing the risk of falls in older adults [14]. Resistance exercise and aerobic exercise are the most commonly used exercises to improve physical function [15, 16]. Studies have confirmed that resistance exercise on land can effectively improve lower limb strength and improve dynamic balance [17, 18]. However, LE may present external fall risk factors such as uneven walking surfaces, which in turn may further lead to fall-related injuries.

Aquatic exercises (AE) are used as an alternative to LE for older adults who have lower levels of physical activity or neuromuscular disorders that impact their balance function [12, 13]. AE refers to exercise therapy performed in a water environment, by immersion in water to perform targeted therapeutic actions [19]. Since the resistance provided by water helps to slow down movement, AE provides a safer training environment for participants than LE. The benefits of AE are due to the physical properties of water: buoyancy, pressure, resistance to motion, and temperature [21]. When exercising in water, buoyancy counteracts the effects of gravity, which reduces the joint burden, alleviates pain, and promotes relaxation [22]. Studies show that as water level increases, body weight decreases: around 50% at waist level, around 75% at chest level, and around 90% at neck level. [23, 24]. Research also indicated that the ankle located at 1 m water depth experiences 981 Pa of pressure, while the hip, located closer to the surface, is subjected to only 98.1 Pa of pressure [25]. The resistance experienced during AE is also unique because it causes muscles to engage in “isokinetic contractions”, offering an effective way to enhance muscle endurance and strength [20]. Due to the resistance from all sides when moving in the water, people will feel obvious obstruction when moving in the water. It takes six times more force to complete the same training program at the same speed in water than on land [26]. Another crucial advantage of AE is the temperature of the water, which is typically maintained at 32–34 ℃ in rehabilitation pools; this temperature promotes blood circulation and further relaxes muscles, aiding the therapeutic process [27]. Overall, the unique properties of water (e.g. buoyancy, hydrostatic pressure, etc.) create an environment where balance is constantly challenged, and that AE requires greater stabilization efforts due to resistance and turbulence than LE, thus engaging more muscle groups and enhancing proprioceptive feedback [20].

Previous systematic reviews have mostly focused on the effects of AE on muscle strength, mobility, and various [28] in older adults [19, 29, 30]. In particular, regarding balance function, some studies have found that AE can better improve lower limb function in older adults [31–33]. However, there are also studies with the opposite conclusion, suggesting that LE may be more effective than AE in improving balance function in older adults [34–37]. Therefore, there is no definitive conclusion on whether AE is superior to comparable LE in improving balance function in older adults. Additionally, due to the different mechanisms of balance dysfunction caused by the central and peripheral nerve systems, there are variations in the effects of AE in improving balance ability [38]. Older adults are confronted with a continuously challenging environment, making balance ability crucial. Therefore, it is necessary to identify the most targeted exercise methods to improve balance function. The purpose of this systematic review is to compare the effects of AE and LE on the balance function of older adults with different health conditions.

## Methods

### Search strategy

This systematic review and meta-analysis study was registered (PROSPERO CRD42023429557) and conducted by the Preferred Reporting Items for Systematic Review and Meta-Analysis (PRISMA) guidelines [39]. Four electronic databases, including PubMed, Web of Science, Scopus, and Embase, were searched from January 2003 to June 2023. The following Medical Subject Headings (MeSH) terms and their synonyms were used either singularly or in combination: “aquatic therapy”, “aquatic exercise”, “water therapy”, “water exercise”, “water-based exercise”, “aquatic physiotherapy”, “aquatic rehabilitation”, “hydrotherapy”, “Ai Chi therapy”, “older”, “aged”, “aging”, “elderly”, “senior”, “balance”, “posture balance”, and “posture control”. The complete search strategy is presented in Supplementary 1.

### Eligibility criteria

Inclusion criteria for this study were defined according to the PICOS approach: 1) Participants: adults aged 60 years or older were included. There was no restriction on the injury or disorder type. 2) Intervention: Studies that utilized various types of AE with clear intervention details, including duration, frequency, type, and intensity, were included. 3) Control: the control group received LE treatment. 4) Outcomes: studies must have reported at least one outcome related to balance and compared the outcomes between the AE and the LE groups. 5) Study design: studies were limited to randomized control trials (RCTs). Only studies that had full-text articles in English were included in this study.

### Study selection and data extraction

To begin the screening process, all records were imported into reference management software (Endnote X9), and duplicate records from the same trial were subsequently removed. Two reviewers (YD and ZT) independently identified studies that potentially met the inclusion criteria and disregarded irrelevant reports. Full-text studies that met the inclusion criteria were obtained for further evaluation. Any disagreements were resolved through discussion, and if necessary, a final decision was made in consultation with a third author (ZTY). Using a standard extraction form developed for this study, both two reviewers (YD and ZT) independently extracted information on participants’ characteristics (e.g., demographics), intervention details (e.g., modality, intensity, frequency, and duration), and balance-related outcomes. In cases where missing data were identified, we made efforts to contact the authors to obtain additional data. Any disagreement in data extraction was resolved through discussions between the reviewers. If disagreements persisted, a final decision was determined in consultation with a third author (ZTY).

### Quality assessment

The methodological quality of the included studies was assessed by two independent researchers (YD and ZT) using the Physiotherapy Evidence Database (PEDro) scale with a maximal score of 10 [40]. The scale assesses the following eleven items: eligibility criteria; random allocation; concealment allocation; baseline similarity; and blinding of the subjects, therapists, and assessors. Each item was rated 0 (item did not meet the criteria) to 1 (item met the criteria) for each study. The higher the total score is, the higher the quality of the study. If disagreements persisted, a final decision was determined in consultation with a third author (ZTY).

### Publication bias assessment

Publication bias can be visually displayed using funnel plots, which allow for the examination of small study effects and the assessment of funnel plot asymmetry through statistical testing [41]. In the absence of publication bias, the funnel plot should exhibit a symmetrical shape, with smaller studies scattered widely at the bottom and larger studies more tightly spread [42].

### Meta-analysis

The meta-analysis was performed using Review Manager (RevMan 5.3). The effect size was calculated as the standardized mean difference (SMD) and 95% confidence intervals (CI). In this study, an SMD of 0.2–0.5 was considered small, 0.5–0.8 medium, and >  = 0.8 a large effect. For all analyses, we used an inverse-variance weighted random-effect model that incorporates heterogeneity into the model if needed. A random-effected model was applied if heterogeneity (I^2^) was more than 50%, and high I^2^ was expected due to the combination of different outcome measures and different populations. To investigate the clinical heterogeneity according to the different measurement instruments and different populations, subgroup analyses were performed. Sensitivity analysis was performed to explore the stability of the results by removing one trial with a distinctly different direction of change in each category of balance-related outcomes. All data were continuous variables and *p* < 0.05 was considered statistically significant.

## Results

### Study selection

**Fig. 1:**
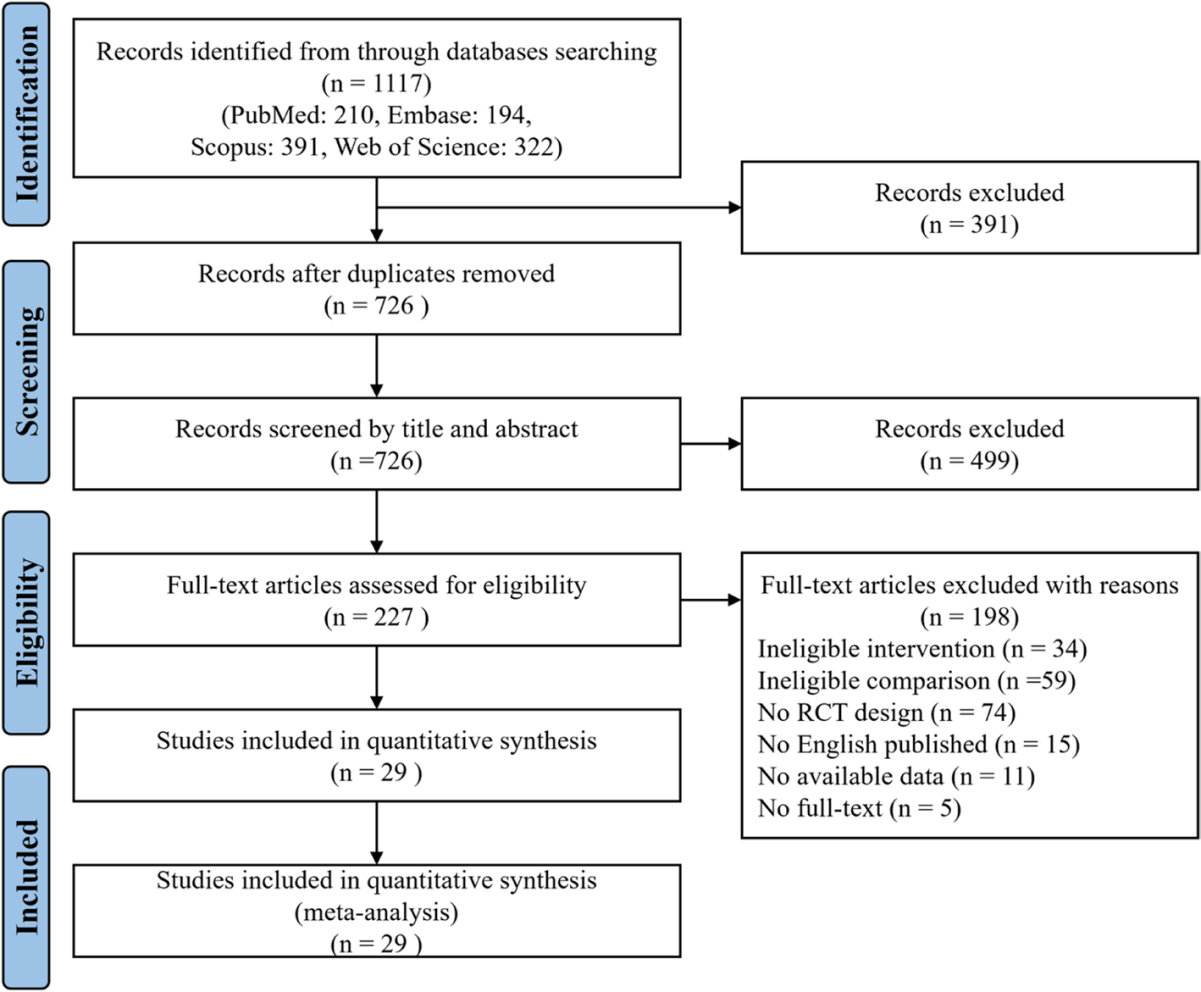
Flow diagram of the study selection process according to Preferred Reporting Items for Systematic Reviews and meta-analysis (PRIAMA)

In total, 1117 potential studies were searched from four electronic databases. Of these studies, a total of 391 duplicate studies were removed, and 499 studies were excluded after screening the titles and abstracts. Then, we obtained the full text of the remaining 227 studies. Furthermore, 198 studies were excluded because they did not meet the eligibility criteria. Finally, we included 29 studies that met the inclusion and exclusion criteria in this systematic review meta-analysis (see Fig. 1).

### Study characteristics

The characteristics of the included studies are shown in Table 1. Twenty-nine studies included in this systematic review were RCTs, that compared the effects of AE and LE on balance in older adults. Among them, 6 studies included healthy subjects [43–48], 9 studies included patients with musculoskeletal disorders [31, 36, 37, 49–54], 5 studies included patients with Parkinson’s disease [33, 55–58], 2 studies included patients with stroke [32, 59], 3 studies included patients with chronic obstructive pulmonary disease (COPD) [35, 60, 61], and 2 studies included patients with heart failure (HF) [34, 62]. In addition, 1 study included coronavirus disease 2019 (COVID-19) patients [63] and 1 study included sedentary lifestyle subjects [64].

**Table 1:** Summary of included studies

Study	Diagnosis			Intervention/Comparison	Sample size pre (post)	Age (mean ± SD)	Water depth	T (℃)	Intervention Period			Outcome measures
									**Min/session**	**Time/week**	**Total duration**	
Bento et al., 2012 [43]	Healthy populations	Healthy		AE	27 (24)	65.6 ± 4.2	Xiphoid level	28–30	60	3	12	6 MWT / 30 CST
				LE	20 (14)	65.6 ± 4.4						
Bento-Torres et al., 2019 [44]		Healthy		AE	14 (14)	71.2 ± 4.4	/	/	/	3	12	6 MWT / 30 CST
				LE	14 (14)	71.7 ± 4.6						
Bocalini et al., 2010 [45]		Healthy		AE	30 (27)	> 62	/	/	45	3	12	30 CST
				LE	20 (18)							
Oh et al., 2015 [46]		Healthy		AE	40 (34)	74.7 ± 2.9	1.2 m	28	60	3	10	TUGT
				LE	40 (32)	68.2 ± 4.4						
Oh et al., 2021 [47]		Healthy		AE	40 (34)	74.7 ± 2.9	1.2 m	28	60	3	10	TUGT
				LE	40 (32)	72.2 ± 4.4						
Sanders et al., 2013 [48]		Healthy		AE	48 (43)	73.6 ± 13.5	1.0–1.2 m	28–29	45	3	16	30 CST
				LE	18 (17)	72.8 ± 27.4						
Vale et al., 2020 [64]		Sedentary Lifestyle		AE	28 (28)	67.3 ± 1.7	1.3 m	31–33	60	2	16	BBS
				LE	26 (26)	67.3 ± 1.7						
Kurt et al., 2018 [55]	Nervous system diseases	Parkinson		AE	20 (20)	62.4 ± 6.8	1.2 m	32	60	5	5	BBS / TUGT
				LE	20 (20)	63.6 ± 7.2						
Lee et al., 2018 [32]		Stroke		AE	19 (18)	57.6 ± 14.0	/	30–33	30	5	4	BBS
				LE	18 (14)	63.7 ± 11.3						
Pérez et al., 2017 [56]		Parkinson		AE	15 (14)	66.8 ± 5.3	1.1–1.45 m	30	45	2	10	BBS / TUGT
				LE	15 (15)	67.5 ± 9.9						
Pérez et al., 2021 [59]		Stroke		AE	15 (15)	63.8 ± 13.6	1.4 m	34–36	45	2	12	BBS / TUGT
				LE	17 (17)	62.7 ± 13.4						
Silva et al., 2020 [33]	Nervous system diseases	Parkinson		AE	14 (14)	63.1 ± 13.6	/	/	60	2	10	BBS / TUGT
				LE	14 (11)	64.2 ± 13.5						
Volpe et al., 2014 [57]		Parkinson		AE	17 (17)	68.0 ± 7.0	/	/	60	5	8	BBS / TUGT
				LE	17 (17)	66.0 ± 8.0						
Volpe et al., 2017 [58]		Parkinson		AE	15 (13)	70.6 ± 7.8	/	/	60	5	8	BBS / TUGT
				LE	15 (11)	70.0 ± 7.8						
Arnold et al., 2008 [49]	Musculoskeletal diseases	HOA		AE	21 (16)	68.6 ± 5.4	Shoulder to waist	30	50	3	20	BBS
				LE	20 (15)	69.1 ± 6.3						
Arnold et al., 2010 [50]		HOA		AE	27 (19)	74.4 ± 7.5	Chest level	\	45	2	11	6 MWT / BBS/ TUGT / 30 CST
				LE	27 (19)	75.8 ± 6.2						
Assar et al., 2020 [31]		KOA		AE	12 (12)	57.5 ± 6.9	1.3 m	32	90	3	8	BBS
				LE	12 (12)	63.8 ± 7.5						
Hale et al., 2012 [51]		OA		AE	23 (20)	75.7 ± 1.1	1.3 m	28	60	2	12	TUGT
				LE	16 (15)	73.5 ± 1.5						
Kuptniratsaikul et al., 2019 [36]		KOA		AE	40 (40)	62.1 ± 6.4	/	/	30	3	4	6 MWT
				LE	40 (40)	61.7 ± 6.9						
Moreira et al., 2020 [54]		Muscle disorders		AE	75 (60)	70.6 ± 6.0	Xiphoid level	31	45	2	16	BBS
				LE	70 (60)	71.9 ± 7.0						
Murtezani et al., 2014 [37]		Osteoporosis		AE	33 (31)	59.8 ± 6.0	Chest level	30	60	3	40	6 MWT / BBS
				LE	31 (30)	60.7 ± 7.6						
Taglietti et al., 2018 [52]		KOA		AE	31 (31)	67.3 ± 5.9	1.2 m	32	60	2	8	TUGT
				LE	29 (29)	68.7 ± 6.7						
Wang et al., 2011 [53]	Musculoskeletal diseases	KOA	AE		28 (26)	66.7 ± 5.6	/	30	60	3	12	6 MWT
			LE		28 (26)	68.3 ± 5.6						
Adsett et al., 2017	Cardiopulmonary diseases	HF	AE		36 (29)	72.9 ± 8.4	Chest level	33–34	60	1	6	6 MWT/ TUGT
			LE		25 (22)	68.3 ± 11.3						
Caminiti et al., 2011 [62]		HF	AE		11 (11)	67.0 ± 6.0	Xiphoid level	31	60	3	24	6 MWT
			LE		10 (10)	69.0 ± 8.0						
de Castro et al., 2020 [35]		COPD	AE		27 (17)	64.0 ± 8.0	1 m	33	60	3	12	6 MWT / TUGT
			LE		23 (14)	65.0 ± 8.0						
Felcar et al., 2018 [60]		COPD	AE		34 (20)	68.0 ± 8.0	1 m	33	60	3	24	6 MWT
			LE		36 (16)	69.0 ± 9.0						
Ferreira et al., 2022 [63]		COVID-19	AE		26 (24)	70.2 ± 4.2	1.4 m	27–32	60	2	16	TUGT
			LE		26 (25)	71.4 ± 4.6						
Liu et al., 2021 [61]		COPD	AE		16 (14)	65.0 ± 11.0	Xiphoid level	26–30	60	2	12	6 MWT / 30CST
			LE		17 (15)	65.0 ± 8.0						

*AE* aquatic-based exercises, *LE* land-based exercise, *OA* osteoarthritis, *KOA* knee osteoarthritis, *HOA* hip osteoarthritis, *COPD* chronic obstructive pulmonary disease, *COVID-19* coronavirus disease 2019, *BBS* Berg balance scale, *TUGT* time up to go test, *6 MWT* 6-min walking test, *30 CST* 30-s chair stand test, *T* Temperature, *SD* standard deviation

A total of 1486 participants were included, of which 1291 (86.9%) completed the trial in which they were enrolled. The number of participants in each study ranged from 10 to 75. The average age of the participants was 66.2 years. The settings of the AE employed were diverse. The water depth varied from 1 m to 1.4 m or the xiphoid level, and the water temperature ranged between 26 °C and 36 °C. However, 7 studies did not report the aquatic setting [33, 36, 44, 45, 57, 58, 65]. The AE settings exhibited differences across all included studies in regard to the intervention duration (30–90 min), frequency (1–5 sessions per week), and total duration (4–40 weeks). The BBS, TUGT, 6-Minute Walking Test (6 MWT), and 30-s Chair Stand Test (30 CST) were used to measure the balance ability. There were no adverse events reported among the included studies.

### Quality assessment

The scores of each study for the quality assessment are shown in Table 2. Out of a maximum of 10 points, 2 studies scored 5 points [35, 60], 6 studies scored 6 points [31, 34, 49, 50, 56, 62], 16 studies scored 7 points [33, 37, 43–48, 51, 52, 54, 55, 61, 64], and 5 studies scored 8 points [32, 36, 57, 59, 63]. The scores of the included studies ranged from 6 to 8 and were accepted. All studies reported random allocation, baseline similarity, group comparison, and point measures. Because of the environment of the intervention, no study blinded the participants and therapists.

**Table 2:** PEDro criteria and scores of the included studies

Study	Random allocation	Concealed allocation	Baseline Similar	Participant blinding	Therapist blinding	Assessor blinding	< 15% dropouts	Intention-to-treat analysis	Group comparison	Point measure	Total (0 to 10)
Adsett et al., 2017 [30]	1	0	1	0	0	1	0	1	1	1	6
Arnold et al., 2008 [49]	1	1	1	0	0	1	0	0	1	1	6
Arnold et al., 2010 [50]	1	1	1	0	0	1	0	0	1	1	6
Assar et al., 2020 [31]	1	0	1	0	0	0	1	1	1	1	6
Bento et al., 2012 [43]	1	1	1	0	0	1	1	0	1	1	7
Bento-Torres et al., 2019 [44]	1	1	1	0	0	1	1	0	1	1	7
Bocalini et al., 2010 [45]	1	1	1	0	0	1	1	0	1	1	7
Caminiti et al., 2011 [62]	1	0	1	0	0	0	1	1	1	1	6
de Castro et al., 2020 [35]	1	0	1	0	0	1	0	0	1	1	5
Felcar et al., 2018 [60]	1	0	1	0	0	1	0	0	1	1	5
Ferreira et al., 2022 [63]	1	1	1	0	0	1	1	1	1	1	8
Hale et al., 2012 [51]	1	1	1	0	0	1	1	0	1	1	7
Kuptniratsaikul et al., 2019 [36]	1	1	1	0	0	1	1	1	1	1	8
Kurt et al., 2018 [55]	1	1	1	0	0	1	1	0	1	1	7
Lee et al., 2018 [32]	1	1	1	0	0	1	1	1	1	1	8
Liu et al., 2021 [61]	1	1	1	0	0	1	1	0	1	1	7
Moreira et al., [54]	1	0	1	0	0	1	1	1	1	1	7
Murtezani et al., 2014 [37]	1	1	1	0	0	1	1	0	1	1	7
Oh et al., 2015 [46]	1	1	1	0	0	1	0	1	1	1	7
Oh et al., 2021 [47]	1	1	1	0	0	1	0	1	1	1	7
Pérez et al., 2017 [56]	1	0	1	0	0	1	1	0	1	1	6
Pérez et al., 2021 [59]	1	1	1	0	0	1	1	1	1	1	8
Sanders et al., 2013 [48]	1	1	1	0	0	1	1	0	1	1	7
Silva et al., 2020 [33]	1	0	1	0	0	1	1	1	1	1	7
Taglietti et al., 2018 [52]	1	0	1	0	0	1	1	1	1	1	7
Vale et al., 2020 [64]	1	1	1	0	0	1	1	0	1	1	7
Volpe et al., 2014 [57]	1	1	1	0	0	1	1	1	1	1	8
Volpe et al., 2017 [58]	1	1	1	0	0	1	1	0	1	1	7
Wang et al., 2011 [53]	1	1	1	0	0	1	1	0	1	1	7

PEDro, the Physiotherapy Evidence Database Scale

### Publication bias assessment

**Fig.2:**
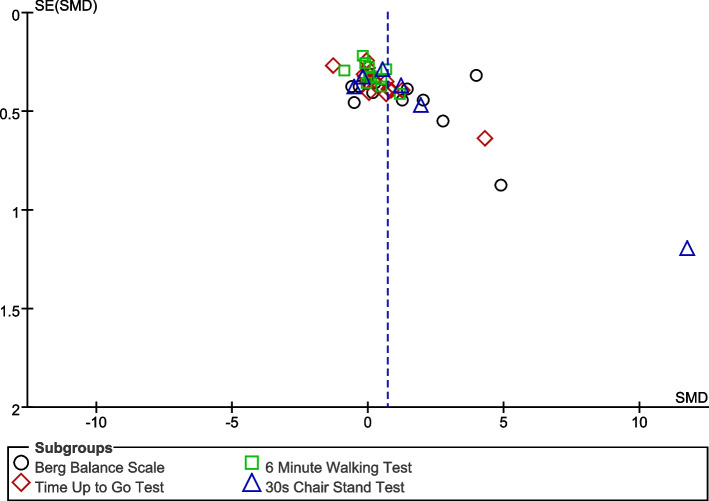
Funnel plot for all the meta-analyses

The visual inspection of the funnel plot identified substantial asymmetry, indicating the possibility of publication bias in the meta-analysis (Fig. 2). Among them, there were 5 studies with significant heterogeneity, including one study on a healthy population [44], two studies on Parkinson’s diseases [56, 57], and two studies on osteoarthritis [49, 52]. Furthermore, BBS was used as the outcome measurement in three studies [49, 56, 57], TUGT was used as the outcome measurement in one study [52], and 30 CST was used as the outcome measurement in one study [44]. Studies using 6 MWT, as well as studies on cardiopulmonary diseases, had acceptable publication bias.

### Balance-related outcomes

**Fig.3:**
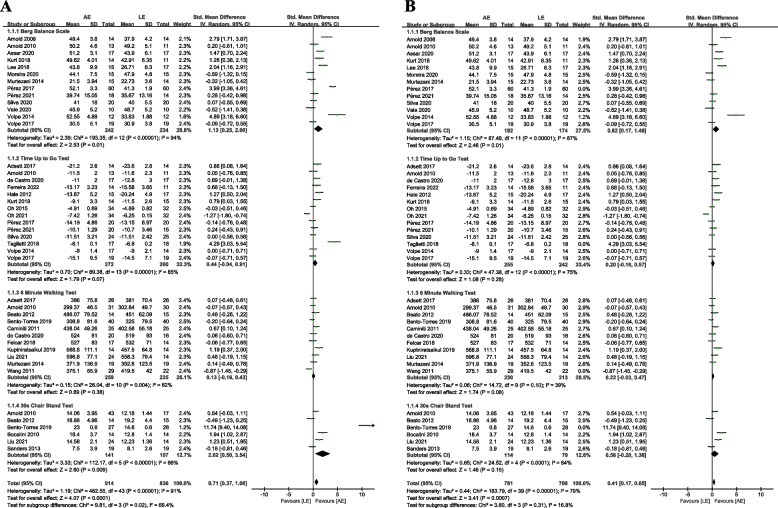
Meta-analysis (**A**) and sensitivity analysis (**B**) of the aquatic-based exercise (AE) versus the land-based exercise (LE) on balance performance. CI: confidence interval; SD: standard deviation

Thirteen studies assessed the effects of resistance training on BBS. A total of data were extracted for 476 participants (AE group, *n* = 242; LE group, *n* = 234). Compared with the LE group, there was a significant increase in BBS in the AE group (SMD = 1.13, 95% CI 0.25 to 2.00, *p* = 0.01, I^2^ = 94%). Eleven studies assessed the effects of resistance training on the 6 MWT. A total of data were extracted for 494 participants (AE group, *n* = 259; LE group, *n* = 235). The results indicated that there was no significant difference between the AE group and the LE group on 6MWT (SMD = 0.13, 95% CI -0.16 to 0.43, *p* = 0.38, I^2^ = 62%). Fourteen studies assessed the effects of resistance training on the TUGT. A total of data were extracted for 532 participants (AE group, *n* = 272; LE group, *n* = 260). The results indicated that there was no significant difference between the AE group and LE group on TUGT (SMD = 0.44, 95% CI -0.04 to 0.91, *p* = 0.07, I^2^ = 85%). Six studies assessed the effects of resistance training on 30 CST. A total of data were extracted for 248 participants (AE group, n = 141; LE group, *n* = 107). Compared with the LE group, there was a significant increase in 30 CST in the AE group (SMD = 2.02, 95% CI 0.50 to 3.54, *p* = 0.009, I^2^ = 96%). See Fig. 3A.

### Subgroup analysis

**Fig.4:**
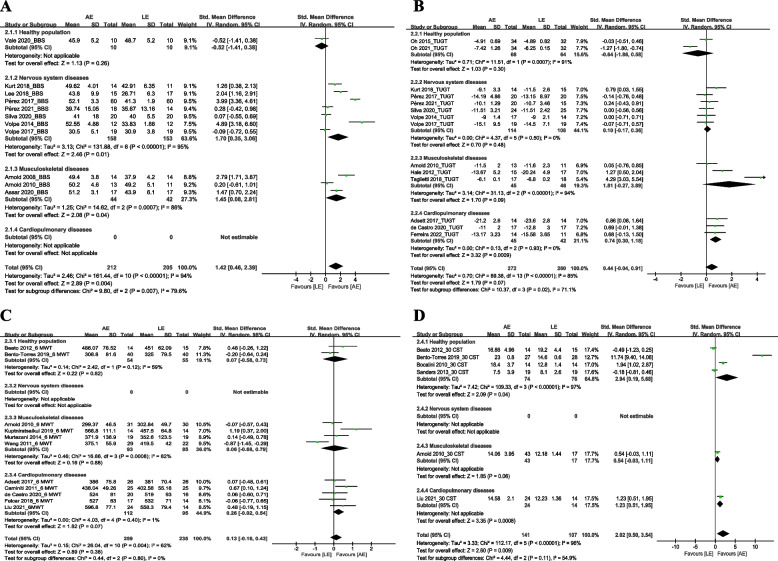
Meta-analysis of the aquatic-based exercise (AE) versus the land-based exercise (LE) on different balance outcomes according to different populations. **A**: Berg balance scale; **B**: Time up to go test; **C**: 6-min walking test; **D**: 30-s chair stand test; BBS, Berg balance scale; TUGT, time up to go test; 6 MWT, 6-min walking test; 30 CST, 30-s chair stand test; CI: confidence interval; SD: standard deviation

To conduct subgroup analysis (Fig. 4), we categorized the included participants into four subgroups: healthy population (healthy and sedentary lifestyle), nervous system diseases (Parkinson’s disease and stroke), musculoskeletal diseases (osteoarthritis, osteoporosis, and muscle disorders), and cardiopulmonary diseases (COPD, HF, and COVID-19). BBS (Fig. 4A): Subgroup analysis showed that there were significant differences between the AE group and the LE group in the population with nervous system diseases (SMD = 1.70, 95% CI 0.35 to 3.06, *p* = 0.01, I^2^ = 95%) and musculoskeletal diseases (SMD = 1.45, 95% CI 0.08 to 2.81, *p* = 0.04, I^2^ = 86%). TUGT (Fig. 4B): Subgroup analysis showed that there a significant difference between the AE group and the LE group in population with cardiopulmonary diseases (SMD = 0.74, 95% CI 0.30 to 1.18, *p* = 0.0009, I^2^ = 0%). 6 MWT (Fig. 4C): Subgroup analysis showed that there was no significant difference between the AE group and the LE group in all population. 30 CST (Fig. 4D): Subgroup analysis showed that there were significant differences between the AE group and the LE group in the healthy population (SMD = 2.94, 95% CI 0.19 to 5.68, *p* = 0.04, I^2^ = 97%) and population with cardiopulmonary diseases (SMD = 1.23, 95% CI 0.51 to 1.95, *p* = 0.0008).

### Sensitivity analysis

The sensitivity analyses after excluding trials with a distinctly opposite direction of change in each category presented that the point estimates changed by -0.31 (SMD = 0.82, 95% CI 0.17 to 1.48, *p* = 0.01, I^2^ = 87%) in the BBS, by -0.24 (SMD = 0.20, 95% CI -0.16 to 0.57, *p* = 0.28, I^2^ = 75%) in the TUGT, by 0.09 (SMD = 0.22, 95% CI -0.03 to 0.47, *p* = 0.08, I^2^ = 39%) in the 6 MWT, and by -1.44 (SMD = 0.58, 95% CI -0.20 to 1.36, *p* = 0.15, I^2^ = 84%) in the 30 CST (Fig. 3B).

## Discussion

With the increase of age and the influence of various chronic diseases, the physical function of older adults decreases significantly. Older adults’ ability to accurately control body movements is limited due to the reduced central nervous system’s ability to process information and significant degenerative changes in skeletal muscles, which ultimately leads to weakened balance ability [66]. Balance plays a crucial role in the daily activities of older adults [64]. The purpose of this meta-analysis was to compare the impact of AE and LE on balance in older adults. The results of our study indicated that AE had a more significant improvement in balance than LE. However, because the health status of the old individuals in the included studies varied, these results must be interpreted with caution.

Balance, coordination, and agility are often used to evaluate physical activity [67]. Balance dysfunction can lead to an increased risk of falls among older adults, subsequently raising mortality and disability rates [68]. A system review revealed that exercise can prevent falls in community-dwelling older people, and exercise programmes that challenge balance and are of a higher dose have larger effects [68]. Youngwook et al., found that both AE and LE intervention demonstrated similar effects on dynamic balance in individuals aged 65 years or older, and offered evidence supporting the use of AE as a viable substitute for LE in enhancing dynamic balance and potentially mitigating the risk of falls [13]. Moreira et al. demonstrated that compared to the LE intervention, AE intervention can be used as a preventive approach for the older adults at risk of falling, to enhance proprioception and increase awareness of fall risk [54, 69]. Patients with Parkinson’s disease and stroke have obvious gait problems [70]. When patients walk training in the water, the standing phase of the lower limbs of the affected side is prolonged due to the support of buoyancy, and the lower limbs of the unaffected side can relatively fully hip flexion, step, and buoyancy can reduce the difficulty of the lower limbs of the affected side in stride hip flexion, and improve gait symmetry [20]. Veldema et al. demonstrated that compared with LE interventions, AE showed superior effects in balance, walking, muscular strength, and cardiorespiratory fitness in patients with stroke [71]. In this study, BBS, 6 MWT, 30 CST, and TUGT are mainly used as indicators to evaluate balance ability. However, the subgroup analysis of our study found that compared to the LE group, AE can only improve BBS in patients with nervous system diseases. AE may offer a more suitable exercise option for older individuals with health conditions compared to LE. Bartels et al. indicated that AE has clinically relevant effects on patient-reported pain and disability in people with KOA and HOA compared to no intervention [19]. However, our subgroup analysis results revealed that AE only significantly improved BBS in patients with musculoskeletal disorders, and there was significant heterogeneity. This may be associated with musculoskeletal disorders that predispose to pain, thereby affecting dynamic balance function test results (i.e. TUGT, 6 MWT, and 30 CST). The tests mentioned, namely BBS are commonly used to evaluate balance ability. However, it is important to note that no single test can comprehensively assess all aspects of balance function. They may not fully capture all dimensions of balance function, such as anticipatory postural adjustments, reactive postural control, or balance during complex tasks. Additionally, individual factors, such as fear of falling or cognitive impairments, can influence test outcomes. Therefore, a comprehensive assessment of balance function may require a combination of different tests, clinical judgment, and consideration of individual factors.

A recent review indicated that AE is an effective physical intervention to enhance physical fitness in healthy adults and adults with chronic diseases [72]. Comparison of balance challenges encountered in AE and LE revealed that LE may focus on static balance exercises, such as standing on one foot, whereas AE may involve dynamic movements, such as walking in water currents or maintaining stability on unstable surfaces, such as aquatic platforms. It was emphasized how the sensory feedback and proprioceptive demands differ between the two modalities, with the AE requiring adaptation to the unique stimuli of the aquatic environment. In the same way, the results of our study found that compared to the LE group, AE had more effects in improving balance ability in older adults with various health conditions. Further, the results of the present study indicated that AE could improve balance ability based on the BBS and the 30 CST. However, there were no significant differences between the AE group and the LE group in terms of the 6 MWT and TUGT. This heterogeneity may be caused by different populations. Subgroup analysis results of our study also found that compared to the LE group, the effects of the AE group on the improvement of balance function in patients with cardiopulmonary diseases was significant, and the heterogeneity was acceptable. The reason for the improved physical function in a water environment may be that the shift in the center of gravity induces more controlled movement and contributes to balance control during the task [21]. As individuals age, their balance and stability naturally decline due to factors such as decreased muscle strength and coordination. By incorporating exercises that offer a higher challenge, such as those performed on unstable surfaces or with dynamic movements, older adults can improve their balance and stability more effectively [54]. By progressively increasing the challenge level of AE exercises, older adults can continue to make gains in their balance and prevent stagnation [20]. Regularly exposing the body to new and more difficult balance demands helps to promote adaptation, strengthen muscles, and enhance the body’s ability to maintain balance in various real-life situations [26]. Older adults often have age-related conditions or disabilities that further compromise their balance. These may include conditions like osteoarthritis [19], Parkinson’s disease [56], or stroke [32].

Providing a higher balance challenge through AE exercises can help stimulate the neuromuscular system, enhance the awareness of body position in space, and improve overall balance control, which is particularly beneficial for individuals with compromised balance abilities [21]. However, it’s critical to determine if these benefits transfer to LE training. Factors like task specificity, environmental similarities, and individual characteristics influence transfer effects [73]. If AE exercises resemble dry land balance testing, transfer effects are likely. The importance of applying the principle of specificity to interventions aimed at improving balance ability was emphasized by Grabiner et al. [74]. Kim et al. found that specific types of balance exercises had limited transfer effects to untrained balance tasks, and that even when these minimal training effects were maintained for several months, the intensity and specificity of the training was properly chosen, despite the relatively small total volume [75]. Further research needs to consider the factors such as specificity, volume, and intensity of the training to maximize the time-effective transfer to real-world scenarios.

### Prospect and limitations

Given the challenges of aging, chronic diseases, and other physiological conditions, older adults must choose a safe method of functional exercise. The findings of this study indicate that AE has a significant improvement over land-LE in improving balance function and can achieve greater safety. Therefore, it is suggested that AE can serve as a preferable alternative to LE. The results of our meta-analysis offer reliable evidence for evaluating the effects of AE. However, several limitations should be acknowledged. First, only English-language studies were included, potentially leading to incomplete representation. Second, the random effects model was employed, revealing significant heterogeneity, likely stemming from variations in exercise types, frequency, duration, and individual health statuses. Thirdly, our study did not focus on the sustainable effects of AE and LE. Our subgroup analysis results have shown that the intervention effects of AE on different health populations are inconsistent (i.e., healthy population, nervous system diseases, musculoskeletal diseases, and cardiopulmonary diseases). However, unfortunately, for some populations, there is insufficient research to confirm this heterogeneity. Finally, there was a difference in the sample size of participants after the AE and LE interventions, and although the sensitivity and subgroup analyses in this study assessed the robustness of the sample size of participants to the results of the meta-analyses to a certain extent, caution should be exercised in interpreting the results of this meta-analyses.

## Conclusion

Although this was influenced by participant health status, transfer effects, sample size, and other factors, AE offers better benefits than LE for improving balance function in older adults.

### Supplementary Information

Supplementary Material 1.

## Data Availability

The data during and/or analyzed during the current study available from the corresponding author.

## Abbreviations

WHO: World Health Organization
BBS: Berg Balance Scale
TUGT: Timed Up and Go Test
LE: Land-based exercises
AE: Aquatic exercises
PRISMA: Preferred Reporting Items for Systematic Review and Meta-Analysis
6 MWT: 6-Minute Walking Test
30 CST: 30-Second Chair Stand Test
