# Supplementary material for: Comparing the effects of aquatic-based exercise and land-based exercise on balance in older adults: a systematic review and meta-analysis
Source: Eur Rev Aging Phys Act. 2024 May 19;21:13. doi: 10.1186/s11556-024-00349-4 (PMC11102618; doi:10.1186/s11556-024-00349-4)
Supplement: Supplementary file 1 — Supplementary Material 1. [file 11556_2024_349_MOESM1_ESM.docx]

**Supplementary materials**

**Search strategy for each database**

1. **PubMed database**

#1 "aquatic therapy"[MeSH Terms] OR "hydrotherapy"[MeSH Terms] OR "balneology"[MeSH Terms] OR "aquatic therapy"[MeSH Terms] OR "aquatic therapy"[MeSH Terms] OR “swimming” [MeSH Terms]

#2"water"[MeSH Terms] AND "seawater"[MeSH Terms] AND " fresh water"[MeSH Terms]

#3"water-based"[All Fields] OR "aquatic*"[All Fields] OR "hydro*"[All Fields]

#4"exercise therapy"[MeSH Terms] OR "physical fitness"[MeSH Terms] OR "sports"[MeSH Terms] OR "exercise movement techniques"[MeSH Terms] OR "gymnastics"[MeSH Terms] OR "education"[MeSH Terms]

#5 #1 AND #2 AND #3 AND #4

#6 "aged"[MeSH Terms] OR aged [MeSH Terms] OR "aging"[MeSH Terms]

#7 aged"[MeSH Terms] OR "aged"[All Fields] OR ("older"[All Fields] AND "adults"[All Fields]) OR "older adults"[All Fields] OR "old"[All Fields] OR ("aged"[MeSH Terms] OR "aged"[All Fields] OR "elderly"[All Fields] OR "elderlies"[All Fields] OR "elderly’s"[All Fields] OR "elderlys"[All Fields]) OR ("aged"[MeSH Terms] OR "aged"[All Fields]) OR ("aging"[MeSH Terms] OR "aging"[All Fields] OR "ageing"[All Fields]) OR "ag"[All Fields] OR ("senior"[All Fields] OR "seniorities"[All Fields] OR "seniority"[All Fields] OR "seniors"[All Fields])

#8 #6 AND #7

#9 (balance[MeSH Terms]) OR (posture[MeSH Terms]) OR (postural Balance[MeSH Terms])

#10 "balance"[All Fields] OR "balanced"[All Fields] OR "balances"[All Fields] OR "balancing"[All Fields] OR "postur*"[All Fields] OR ("controling"[All Fields] OR "controllability"[All Fields] OR "controllable"[All Fields] OR "controllably"[All Fields] OR "controller"[All Fields] OR "controller s"[All Fields] OR "controllers"[All Fields] OR "controlling"[All Fields] OR "controls"[All Fields] OR "prevention and control"[MeSH Subheading] OR ("prevention"[All Fields])

#11 #9 AND #10

#12 randomized controlled trial [pt]

#13 controlled clinical trial [pt]

#14 randomized [tiab]

#15 randomly [tiab]

#16 trial [ti]

#17 #12 OR #13 OR #14 OR #15 OR #16

#18 animals [mh] NOT humans [mh]

#19 #17 NOT #18

#20 #5 AND #8 AND #11 AND #19

1. **Embase**

#1 ('aged'/exp OR 'aging'/exp OR 'elderly'/exp OR senior OR 'old age'/exp)

#2 ('aged'/exp OR aged OR aging:ab,ti OR aged:ab,ti OR elderly:ab,ti OR senior:ab,ti)

#3 #1 AND #2

#4 (balance OR 'balance impairment'/exp OR 'balance disorder'/exp OR 'postural balance'/exp OR 'body equilibrium'/exp)

#5 ('balance disorder':ab,ti OR balance:ab,ti OR 'postural balance':ab,ti OR 'body equilibrium':ab,ti)

#6 #4 AND #5

#7 ('aquatic exercise'/exp OR 'water sport'/exp OR 'hydrotherapy'/exp OR 'aquatic sports'/exp)

#8 (‘exercise’/exp or ‘movement’/exp or ‘locomotion’/exp or ‘physical exertion’/exp or ‘exercise therapy’/exp or ‘physical endurance’/exp or ‘physical fitness’/exp or ‘sports’/exp or ‘exercise movement techniques’/exp or ‘fitness centers’/exp or ‘physical therapy modalities’/exp or ‘rehabilitation’/exp or ‘gymnastics’/exp)

#9 ((‘water’ or ‘water-based’ or ‘seawater’ or ‘aqua’ or ‘aquatic$’ or ‘hydrokinetic$’ or ‘hydrokinetic$’ or ‘pool’ or ‘pool-based’ or ‘swimming pool’) adj10 (‘exercise$’ or ‘fitness’ or ‘physiotherap$’ or ‘activit$’ or ‘aerobic’ or ‘training’ or ‘therap$’ or ‘rehabilitation’ or ‘treadmill’ or ‘walking’ or ‘gymnastic$’ or ‘calisthenic$’)):ti:ab

#10 ‘treading water’ or ‘swimming’ or ‘swim’ or ‘aquarobics’ or ‘aquatone’ or ‘Ai Chi’ or ‘Halliwick’ or ‘hydrotherap$’ or ‘whirlpool bath$’):ti:ab

#11 #7 AND #8 AND #9 AND #10

#12 ‘randomized controlled trial’:ti,ab

#13 controlled clinical trial’:ti,ab

#14 ‘randomized’:ti:ab

#15 clinical trials as topic’/exp

#16 ‘randomly’:ti:ab

#17 “trial”:ti

#18 #12 OR #13 OR #14 OR #15 OR #16 OR #17

#19 animals /exp NOT humans /exp

#20 #18 NOT #19

#21 #3 AND #6 #11 AND #20

1. **Scopus**

((ALL(aquatic exercise) OR ALL(aquatic therapy) OR ALL(water therapy) OR ALL(water exercise) OR ALL(aquatic physiotherapy) OR ALL(aquatic rehabilitation) OR ALL(hydrotherapy) OR ALL(balneotherapy) OR ALL(immersion therapy) OR ALL(hydrokinesitherapy)) AND ((TITLE -ABS(aquatic exercise) OR TITLE-ABS(aquatic therapy) OR TITLE-ABS(water therapy) OR TITLE-ABS(water exercise) OR TITLE-ABS(aquatic physiotherapy) OR TITLE-ABS(aquatic rehabilitation) OR TITLE-ABS(hydrotherapy) OR TITLE-ABS(balneotherapy) OR TITLE-ABS(immersion therapy) OR TITLE-ABS(hydrokinesitherapy)))

AND

((TITLE-ABS(aged) OR TITLE-ABS(aging) OR TITLE-ABS(elderly) OR TITLE-ABS(senior) OR TITLE-ABS(old age)) AND (ALL(aged) OR ALL (aging) OR ALL(elderly) OR ALL(senior) OR ALL(old age)))

AND

((TITLE-ABS(balance) OR TITLE-ABS(postural balance) OR TITLE-ABS(balance control) OR TITLE-ABS(postural control)) AND ALL(balance) OR ALL(postural balance) OR ALL(balance control) OR ALL(postural control)))

AND

((TITLE-ABS(randomized controlled trial) OR TITLE-ABS(controlled clinical trial) OR TITLE-ABS(randomized)) AND ALL(randomized controlled trial) OR ALL(controlled clinical trial) OR ALL(randomized)))

1. **Web of Science**

#1 (((((((TS=(aquatic exercise)) OR TS=(aquatic sports)) OR TS=(aquatic therapy)) OR TS=(water therapy)) OR TS=(water exercise)) OR TS=(aquatic physiotherapy)) OR TS=(aquatic rehabilitation)) OR TS=(balneotherapy)) OR TS=(immersion therapy)) OR TS=(hydrotherapy)

#2 (((((((TI=(aquatic exercise)) OR TI=(aquatic sports)) OR TI=(aquatic therapy)) OR TI=(water therapy)) OR TI=(water exercise)) OR TI=(aquatic physiotherapy)) OR TI=(aquatic rehabilitation)) OR TI=(balneotherapy)) OR TI=(immersion therapy)) OR TI=(hydrotherapy)

#3 #1 AND #2

#4 ((((TS=(aged)) OR TS=(aging)) OR TS=(elderly)) OR TS=(senior)) OR TS=(older)

#5 ((((TI=(aged)) OR TI=(aging)) OR TI=(elderly)) OR TI=(senior)) OR TI=(older)

#6 #4 AND #5

#7 (((TS=(balance)) OR TS=(postural control)) OR TS=(postural balance)) OR TS=(balance control)

#8 (((TI=(balance)) OR TI=(postural control)) OR TI=(postural balance)) OR TI=(balance control)

#9 #7 AND #8

#10 (((TS=(randomized controlled trials)) OR TS=(controlled clinical trial)) OR TS=(randomized)) OR TS=(randomized controlled trial)

#11 #3 AND #6 #9 AND #10
